# The role of *Astragalus membranaceus* and its derivatives in digestive system diseases: reconstruction of immune tolerance

**DOI:** 10.3389/fimmu.2026.1855390

**Published:** 2026-06-17

**Authors:** Xinyue Chang, Xin Zhang, Hanqing Guo, Xinqi Yao, Jun Zhang, Kun Zhuang, Lin Li

**Affiliations:** 1 Department of Gastroenterology, Xi’an Central Hospital, Xi’an, Shaanxi, China; 2 Department of Microbiology and Immunology, School of Basic Medical Sciences, Shanxi Medical University, Taiyuan, Shanxi, China

**Keywords:** *Astragalus membranaceus*, digestive system diseases, immune regulation, immune tolerance, traditional Chinese medicine

## Abstract

The gastrointestinal tract, a vital immune organ in the human body, serves as the primary site of direct contact with external antigens. Maintaining immune tolerance is essential for preventing the onset and progression of digestive system diseases. Autoimmune diseases and tumors represent the overactivation and evasion of the immune system, respectively, and the imbalance of “immune tolerance” is the common core mechanism underlying both conditions. The function of traditional Chinese medicine in regulating the immune function of the digestive system has attracted wide attention, especially in the dual regulation of immune tolerance. *Astragalus membranaceus* and its derivatives exhibit remarkable efficacy in restoring immune tolerance. Mechanistically, these agents exert multi-target regulatory effects via modulating core signaling pathways, remodeling immune cell function and gut microbiota composition. Concurrently, they rebalance pivotal immune axes, such as Th1/Th2 and Treg/Th17, thereby systematically reinstating immune homeostasis rather than exerting isolated, unilateral modulations. This review systematically summarizes 112 studies published in PubMed, Web of Science and CNKI from December 2020 to December 2025, focusing on the immunomodulatory mechanisms and evidence of *Astragalus membranaceus* and its derivatives in the treatment of digestive diseases. It further examines the limitations of current studies and potential future research directions, thereby offering a theoretical basis for the development of novel drugs derived from *Astragalus membranaceus* and its active compounds.

## 1 Introduction

Immune tolerance refers to a state of antigen-specific immunological unresponsiveness, particularly toward self-antigens or harmless foreign antigens. It underpins the immune system’s capacity to discriminate between “self” and “non-self” (1), which allows the immune system to act precisely, killing pathogens or cancer cells while avoiding damage to normal tissues (2). If immune tolerance is lost, the immune system may attack the “self,” resulting in autoimmune diseases. Conversely, excessive or inappropriate immune tolerance may prevent the immune system from responding to harmful “non-self” substances, leading to chronic infections and tumors (3–5). Therefore, immune tolerance is the cornerstone for maintaining a stable internal environment and immune homeostasis.

The gastrointestinal tract is recognized as a part of the human body’s largest immune system, which is capable of not only defending against invasive pathogens but also maintaining immune tolerance toward non-pathogenic antigens such as those derived from food (6, 7). This dynamic balance between “acceptance” and “defense” is vital for preserving intestinal homeostasis (8, 9). Thus, the regulation of immune tolerance is recognized as a key and multifaceted player in digestive diseases. Concerning digestive diseases caused by immune tolerance disorders, such as inflammatory bowel disease (IBD), celiac disease, food allergies, and neoplasms, current treatments mainly aim to control inflammation and relieve corresponding symptoms, yet fail to solve the fundamental problems of the immune dysregulation (10). In contrast, traditional Chinese medicine (TCM) constitutes a different treatment framework.

Within this conceptual framework, the evolution from classical herbal compatibilities to modern bioactive extracts and innovative formulations has emerged as a pivotal trajectory in the modernization of TCM. *Astragalus membranaceus* (AM) has garnered significant attention owing to its profound theoretical foundations and well-defined translational potential in modern medicine. According to TCM theory, AM can stabilize the defense line by “Yi Qi Gu Biao” (a TCM strategy for reinforcing qi to stabilize the exterior), eliminate internal pathogenic factors and generate vital energy by “Qu Du Sheng Ji” (a TCM strategy for removing toxins to promote tissue regeneration), and ultimately achieve a state of “balancing Yin and Yang” (11). Furthermore, modern pharmacological investigations have not only validated the utility of AM and its derivatives in the prevention and adjuvant treatment of various related diseases, but also elucidated their characteristic multi-target, multi-pathway regulatory mechanisms at the molecular level (12–14). This mechanism is not simply to inhibit or enhance the immune function, but to restore the disordered immune state to the normal equilibrium point, which is the key to the reconstruction of immune tolerance. Hence, this review seeks to offer a novel perspective on the prevention and management of gastrointestinal disorders by integrating the mechanisms governing immune tolerance with the therapeutic properties of AM and its related compounds.

The literature search utilized keywords related to AM and its active ingredients, including *Astragalus membranaceus* (AM), Astragalus polysaccharide (APS), Astragaloside, Astragalus flavonoid (AF), as well as digestive diseases such as inflammatory bowel disease (IBD), ulcerative colitis (UC), Crohn’s disease, gastritis, colorectal cancer (CRC) and intestinal barrier injury.

Study selection strictly adhered to predefined inclusion and exclusion criteria based on the PICOS framework: (1) Participants: patients or experimental models with digestive diseases; (2) Intervention: AM or its derivatives; (3) Comparator: not restricted; (4) Outcomes: reports on immune tolerance-related indicators; (5) Study design: clinical trials and basic experimental studies. Only full-text articles in English or Chinese were considered. Exclusion criteria encompassed studies with irrelevant topics, data deficiency, and methodological flaws. Ultimately, 112 eligible publications were included for the systematic synthesis of the immunomodulatory mechanisms of AM in the treatment of digestive diseases.

## 2 AM and its derivatives

*Astragalus* is a large genus of leguminous plants with important medicinal value. As a key tonic herb for invigorating qi and strengthening the spleen, its use in China can be traced back thousands of years, and it is now widely used in many countries (15, 16). Recently, research on AM and its derivatives has extended far beyond its traditional applications, demonstrating significant pharmacological effects in immunomodulatory, anti-inflammation, antitumor and so on (17–19).

The pharmacological versatility of *Astragalus* is primarily ascribed to its abundant and varied chemical constituents, including polysaccharides, flavonoids, saponins, alkaloids and different mineral elements, which collectively endow it with multiple pharmacological properties (20, 21). Among these, polysaccharides, flavonoids, and saponins represent its most core active components and serve as the primary material basis mediating its pharmacological effects (22). As a major active component, APS is a water-soluble heteropolysaccharide predominantly composed of monosaccharides such as glucose, fructose, arabinose, and mannose (23). It can effectively activate an array of immune cells and promote the expression of cytokines and chemokines (24, 25). For instance, one study demonstrated that APS enhances CD8^+^ T cell function and inhibits colorectal cancer (CRC) development by regulating the STAT3/galectin-3 (Gal-3)/LAG3 pathway (26). Other studies have indicated that APS may repair intestinal barrier damage by upregulating tight junction protein levels and preventing ferroptosis of intestinal epithelial cells. Furthermore, APS modulates the balance of immune cells, suppresses the production of inflammatory mediators to maintain intestinal immune homeostasis, and regulates the gut microbiota and its metabolites to reshape the intestinal microecology, thereby exerting therapeutic effects in IBD (27). Notably, APS with different molecular weights or structures exhibits distinct mechanisms of action. Specifically, APS with a molecular weight below 10 kDa was shown to alleviate symptoms of UC more effectively than APS ranging from 10 to 50 kDa (28). In summary, APS primarily functions to modulate immune homeostasis and repair the intestinal barrier. Its mechanistic characteristics are highly consistent with and well interpret the traditional TCM theory of “reinforcing qi to stabilize the exterior”. Such consistency further highlights its role in enhancing the body’s immune defense capacity.

In addition to polysaccharides, flavonoids represent another major group of active components in AM. To date, more than 50 flavonoids have been identified in AM, which are powerful natural antioxidants with a variety of pharmacological effects (29–32). The flavonoid fraction of *Astragalus* is dominated by isoflavones and flavonols, exemplified by calycosin and formononetin (FMN). Structurally, these molecules feature a C6–C3–C6 backbone that is susceptible to hydroxylation, methylation, and glycosylation. These substitutions are pivotal in governing the physicochemical properties and pharmacokinetic profiles (e.g., bioavailability) of the compounds (33). Calycosin is one of the most representative isoflavones in *Astragali Radix*. Studies have shown that treatment with *Astragali Radix* total flavone or calycosin significantly increases serum IgA and IgG levels in weaned piglets, improved intestinal morphology and the abundance of goblet cells, and regulated intestinal microbial diversity and composition, thereby reducing the diarrhea rate (34). Similar to Calycosin, FMN is another key isoflavone in AM. On the one hand, it can alleviate the occurrence and development of IBD by activating the MAPK/PPAR-γ/ROS pathway and inhibiting the expression of NLRP3 inflammasome-related proteins (35). On the other hand, FMN inhibits the development of colitis-associated CRC by suppressing the proliferation of colon cancer cells, inducing autophagy and apoptosis pathways, and modulating lipid metabolism (36). These dual functions of FMN reflect its multi-target and multi-pathway properties, which coordinately modulate signaling pathways and cytokines to restore colonic tissue homeostasis. These findings demonstrate that the antioxidant and anti-apoptotic capacities of AFs provide a scientific rationale for the TCM principle of “removing toxins to promote tissue regeneration”.

The third key active ingredient of AM is Astragalus saponins (AS), a class of triterpenoid saponins typically comprising aglycones and sugar moieties linked by glycosidic bonds. The fundamental aglycone scaffold of these compounds is cycloastragenol. Based on variations in their sugar moieties, they can be classified into multiple types, among which the most extensively and deeply investigated one is Astragaloside IV(AS-IV) (37). Current research indicates that AS-IV induces an anti-inflammatory macrophage phenotype, a shift mediated by its regulatory action on the STAT pathway. Meanwhile, AS-IV suppresses the activation of the PI3K/AKT pathway, thereby reducing inflammation and enhancing intestinal epithelial barrier integrity (38, 39). Thus, AS-IV is capable of ameliorating experimental colitis symptoms both *in vitro* and *in vivo*, rendering it a promising candidate for IBD therapy. At the same time, AS-IV has been demonstrated to mitigate the progression of colonic adenomatous polyps (CAP) in mice subjected to a high-fat diet. This effect is mediated by the modulation of intestinal microbiota and metabolomic profiles, as well as by influencing the Wnt3a/β-catenin pathway (40). This indicates a prospective innovative approach to preventing CRC, highlighting the role of the contribution of AS-IV in antitumor effects. Taken together, the core pharmacological action of Astragalosides lies in their anti-inflammatory and antitumor effects, which also provides a direct modern biological interpretation for the traditional TCM efficacy of “removing toxins to promote tissue regeneration”. Mechanistically, these compounds facilitate wound healing and tissue regeneration by suppressing sustained inflammation and resolving microenvironmental barriers.

**Table 1** summarizes the evidence that AM and its derivatives have pharmacological effects on digestive diseases. These components often work in synergy through multiple targets and pathways in the body, jointly forming the modern pharmacological basis for the traditional effects of AM.

**Table 1:** Mechanisms of action of AM and its derivatives on the Digestive system diseases.

Model	AM and its derivatives	Disease	Role of AM and its derivatives	Reference
Mice	APS	CRC	Modulating the STAT3/Gal-3/LAG3 pathway to specifically enhance CD8^+^ T cell function.	(26)
Mice	APS	UC	Regulating Tfh/Treg cell balance and related cytokine expression.	(27)
Mice	APS-G2 (homogeneous α-1,4-glucan backbone)	IBD	Regulation of the SIRT1/PGC-1 α/NF-κB pathway and FXR-mediated signaling via anti-inflammatory and anti-apoptotic actions.	(28)
Mice and cells	APS	UC	Inhibition of the Nrf2/HO-1 pathway prevents ferroptosis in DSS-induced mice and RSL3-stimulated Caco-2 Cells.	(112)
Piglets	Total flavone or Calycosin	Diarrhea	Elevation of serum IgA and IgG levels and improvement of intestinal health via morphology, goblet cells, and microbiota modulation in weaned piglets.	(34)
Mice and cells	FMN	IBD	Inhibition of mitochondrial dysfunction and suppression of NLRP3 inflammasome via MAPK/PPAR-γ/ROS pathway activation and NF-κB nuclear translocation reduction.	(35)
Mice and cells	FMN	Colitis-associated colon cancer	Inhibition of colon cancer cell growth through activation of autophagy/apoptosis and regulation of lipid metabolism.	(36)
Mice	AS-IV	IBD	Regulation of macrophage phenotype via modulation of the STAT signaling pathway.	(38)
Human and Mice	AS-IV	UC	Suppression of inflammation and improvement of intestinal barrier integrity via PI3K/AKT pathway inhibition and microbiota modulation.	(39)
Mice	AS-IV	CAP	Increase the proportion of beneficial intestinal bacteria, suppress the expression of pro-inflammatory cytokines, tumor-associated markers, and Wnt/β-catenin pathway proteins in the colon, and effectively inhibit the proliferation of human colon cancer cell lines (HT29, HCT116, and SW620).	(40)

## 3 Mechanism exploration: how do AM and its derivatives achieve “bidirectional regulation” of immune tolerance in the digestive system?

Although numerous studies have reported the potential of AM and its derivatives for treating various disorders and possible molecular mechanisms, it is still unclear how AM and its derivatives exert dual immune tolerance. This uncertainty poses certain challenges to their clinical translation. Therefore, gastrointestinal immune regulation constitutes a key intersection between traditional TCM practice and modern pharmacological research. An in-depth understanding of this mechanism not only reveals the pathogenesis of tumors, autoimmune diseases, chronic intestinal inflammation and other diseases, but also holds the potential to yield new therapeutic targets and approaches. Here, we organize and analyze the relevant data to clarify how AM and its derivatives exert the “bidirectional regulation” of immune tolerance in the digestive system.

### 3.1 Immunological basis of the digestive system

**Figure 1:**
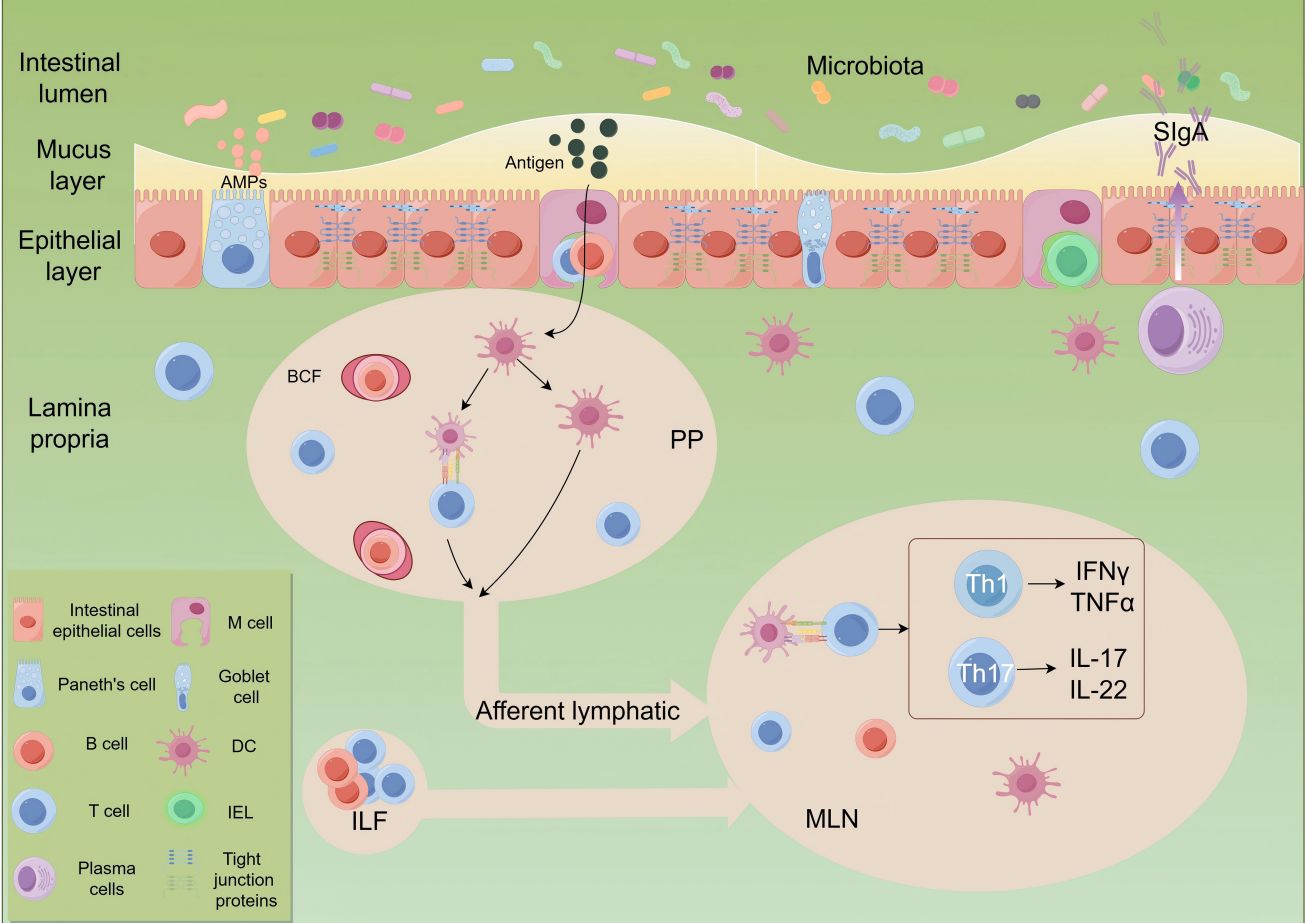
Mucosal immunity of the digestive system. The physical barrier formed by intestinal epithelial cells, goblet cells, tight junction proteins, and the mucus layer secreted by them, together with the chemical barrier formed by gastric acid, bile acid, and antimicrobial peptides, prevents the invasion of foreign pathogens. Gut-associated lymphoid tissues (GALT) primarily comprise Peyer’s patches (PP), isolated lymphoid follicles (ILF), appendix and Waldeyer’s ring, which fulfill a crucial function in antigen recognition and uptake. The mesenteric lymph node (MLN) is the main site of immune induction. Following antigen uptake and processing, antigen-presenting cells actively migrate to MLN through lymphatic vessels and present antigen information to T cells and B cells. Intraepithelial lymphocytes (IEL) exert direct cytotoxic effects through CD8^+^T cells to rapidly and precisely eliminate local threats. Immunoglobulin A (IgA) secreted by lamina propria lymphocytes (LPL), mainly mature plasma cells, can enter the mucosal surface, neutralize antigens and protect the body. CD4^+^ helper T cells, subsets of CD8^+^T cells, regulatory T cells, innate lymphoid cell 3 (ILC3) cells and other cells secrete various cytokines and contribute to immune modulation.

The digestive system is equipped with a sophisticated and unique immune defense and regulatory network. *Astragalus* exerts a beneficial influence on the immune function of the digestive system, with the intestinal tissue—serving as the largest and most pivotal immune organ within this system—representing a primary target of *Astragalus*. Its core mission is to continuously cope with exposure to massive foreign antigens, accurately distinguish between self and non-self and harmless/harmful agents, establish symbiotic tolerance to food antigens, and effectively defend against pathogens (41). This relies on the robust physical and chemical barriers of the digestive tract, its distinctive tissue structure, key immune cells and the gut microbiota (**Figure 1**).

The mechanical barrier includes intestinal epithelial cells, goblet cells, tight junction proteins, and the mucus layer they produce, which prevent the invasion of foreign pathogens, along with the chemical barrier formed by gastric acid, bile acid, antimicrobial peptides, and other components (42–44). Meanwhile, gut-associated lymphoid tissue (GALT), a major and multifaceted component of the mucosa-associated lymphoid tissue (MALT), mainly includes Peyer’s patches (PP), isolated lymphoid follicles (ILF), appendix and Waldeyer’s ring (45). PP fulfills a crucial function in antigen capture and local immunity based on its M cells that take up a variety of antigens in the intestinal lumen (46). ILF is the main component of GALT, in which over 90% of cells are lymphocytes, with a slightly higher proportion of T cells than B cells (47).

The mesenteric lymph nodes (MLNs) serve as the main immune decision-making site. After capturing antigens, intestinal antigen-presenting cells migrate to MLNs via lymphatic vessels and present antigen information to T and B cells, making the final immune decision to attack or tolerate (48–51). Therefore, PP, ILF and MLN are recognized as the “induction sites” of immune responses. Subsequently, the immune command is carried out by the intestinal mucosa epithelium and lamina propria. Intraepithelial lymphocytes (IEL), the largest lymphocyte population in the body, primarily utilize CD8^+^T cells to precisely and rapidly eliminate local threats through direct cytotoxic effects (52).

Mature plasma cells account for the majority of lamina propria lymphocytes (LPLs). These plasma cells secrete abundant IgA, which is then transported to the mucosal surface, neutralizes antigens, and protects the body (53). Secondly, the number of T lymphocytes is also quite large, mainly CD4^+^ helper T cells, a small number of CD8^+^T cells, regulatory T (Treg) cells and innate lymphoid cells. These cells secrete distinct cytokines and play an immunomodulatory role together (54). Mucosal antigen-specific T and B cells can migrate from the initiation site of the immune response to the mucosal effector sites, such as the intestine, differentiate into antigen-specific effector cells, and mediate systemic mucosal immune responses.

Ultimately, the immune function of the digestive system is fundamentally characterized by a complex and dynamic regulatory network arising from the interplay among the epithelial barrier, immune cells, and symbiotic flora. AM and its derivatives achieve their bidirectional immunomodulatory effects precisely by orchestrating this intricate system.

### 3.2 Regulation of immune cells and their functions by AM and its derivatives

**Figure 2:**
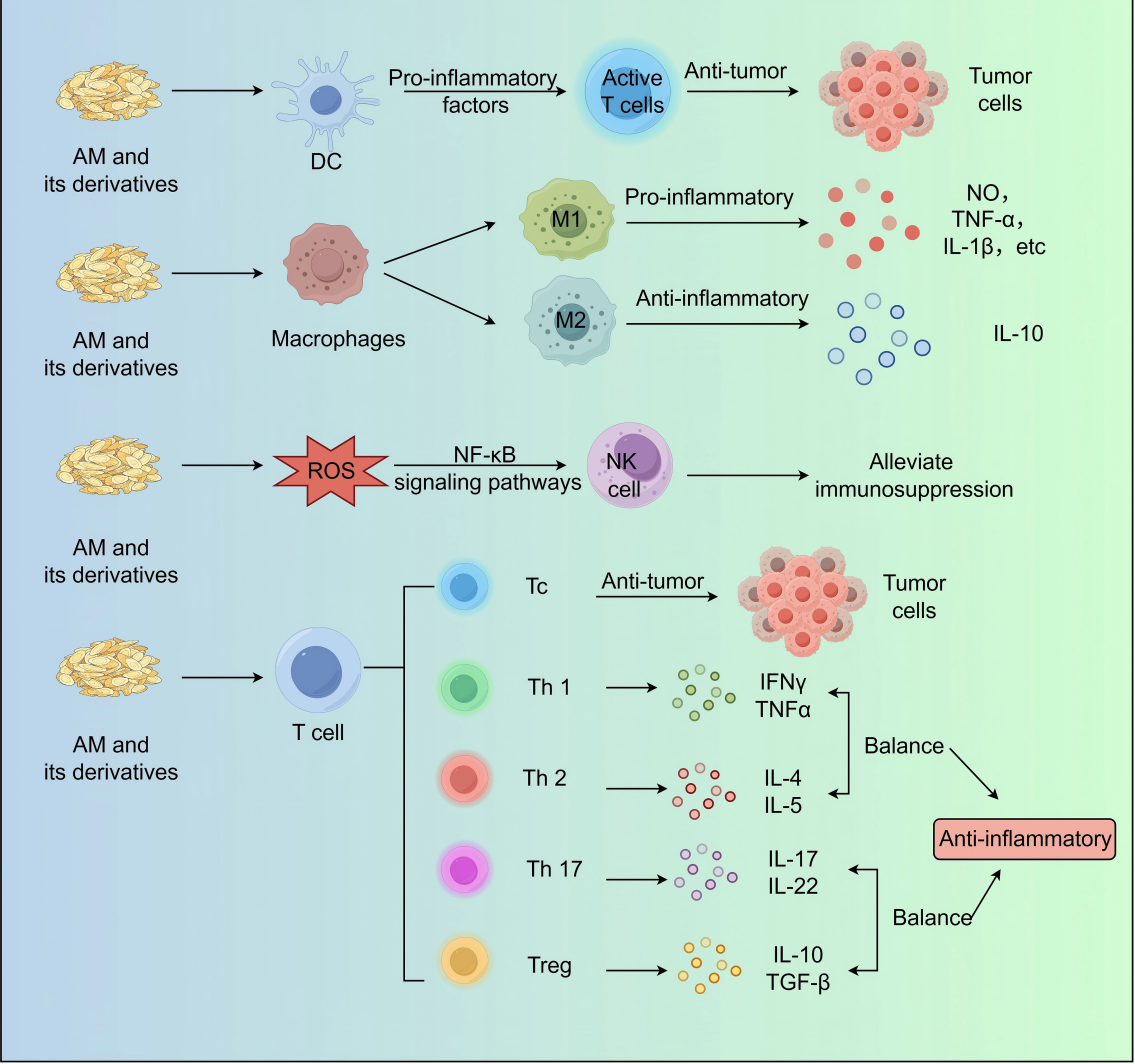
AM and its derivatives regulate immune cells and their functions. The immune cells regulated by *Astragalus membranaceus* (AM) and its derivatives encompass DCs, macrophages, NK cells and T cells. AM and its derivatives can activate DCs and enhance the surface expression of costimulatory molecules on antigen-presenting cells, thereby driving the activation and functional maturation of immune cells. They can also suppress the secretion of pro-inflammatory cytokines triggered by excessive activation of macrophages, thereby alleviating inflammation. AM and its derivatives can promote the M1 polarization, thereby enhancing their phagocytosis ability, antigen presentation ability and pathogen killing function. Alternatively, they can induce the M2 polarization of macrophages, thereby eliciting the production of anti-inflammatory mediators such as IL-10. AM and its derivatives can significantly enhance the activity of NK cells and the phagocytosis of macrophages, potentially through the activation of the NF-κB signaling pathway and increased production of reactive oxygen species (ROS). AM and its derivatives can induce the proliferation and activation of T lymphocytes, such as enhancing the activity of CD8^+^ cytotoxic T lymphocytes (CTL) by down-regulating inhibitory checkpoint molecules such as Tim-3, thereby improving anti-tumor immune function. These compounds help regulate the balance among Th1, Th2, Th17, and Treg cells, contributing to the suppression of inflammatory responses.

AM and its derivatives have become one of the current research hotspots in the field of disease therapy due to their significant immunomodulatory effects on various immune cells. As shown in **Figure 2**, immune cells regulated by AM mainly include macrophages, dendritic cells (DCs), natural killer cells (NK cells), and T cells. APS can activate macrophages and DCs, which can significantly induce the surface expression of costimulatory molecules on antigen-presenting cells, thereby promoting the activation and functional maturation of immune cells (55, 56). Such maturation is essential for efficient antigen presentation and the initiation of adaptive immune responses. In contrast, AM has been shown to suppress the secretion of pro-inflammatory cytokines from overactivated macrophages, thereby attenuating inflammatory responses in UC and other inflammatory disease models (57, 58). This bidirectional regulatory ability of AM may be achieved by modulating associated signal transduction, such as inflammation and cell growth. On the one hand, AM promotes M1 macrophage polarization, enhancing phagocytosis, antigen presentation, and pathogen clearance by inducing the moderate release of proinflammatory mediators (59, 60). On the other hand, it can also induce M2 polarization, stimulate the secretion of anti-inflammatory factors such as IL-10, inhibit excessive inflammation, and participate in tissue repair (24, 42). This modulation of the M1/M2 phenotypic balance is a central manifestation of its bidirectionality.

In addition, as an important component of innate immunity, NK cells also exhibit bidirectionality after treatment with AM and its derivatives. For example, the combination of ginseng, AM and Burar root significantly increased NK cell activity and macrophage phagocytosis, which may be achieved by activating reactive oxygen species and NF-κB signaling pathways (61). However, AS-IV prevents the recruitment of NK cells to the brain after ischemia by inhibiting the chemotaxis mediated by glial cell-derived CCL2. Furthermore, AS-IV can also inhibit the STAT3 signaling pathway and diminish the chemotactic infiltration of NK cells into the brain tissue after ischemia to exert neuroprotective effects in acute ischemic brain injury, offering a potential strategy for immunotherapy of ischemic stroke (62).

The bidirectional immunomodulatory effect of AM on T lymphocytes is one of its most essential and intricate mechanisms. AM has been demonstrated to stimulate the proliferation and activation of T lymphocytes, especially enhancing the activity of CD8^+^ cytotoxic T lymphocytes (CTL) by downregulating inhibitory checkpoint molecules like Tim-3, thereby potentiating antitumor immunity (63, 64). Studies have indicated that administration of *Astragalus* preparations can restore immune balance by modulating the Treg/Th17 cell ratio, thereby alleviating the autoimmune pathology observed in Sjogren’s syndrome and morphine-induced immunosuppression (65, 66). In the experimental autoimmune encephalomyelitis model, Huangqi-Guizhi-Wuwu Decoction inhibited inflammatory infiltration in the central nervous system. It upregulated IL-10 and Foxp3 production by increasing the proportion of Treg cells and downregulated the expression of IFN-γ and IL-17 by reducing the proportion of Th1 and Th17 cells, thereby effectively inhibiting the activation of CD4^+^ T cells (67).

Taken together, these findings confirm that AM and its derivatives can modulate the activities of innate and adaptive immune cells via multiple targets, thereby maintaining the immune homeostasis and strengthening the host defense against various diseases. This characteristic of “guided by the situation and bidirectional regulation” makes AM and its derivatives different from the simple immune “stimulants” or “inhibitors”, which are more in line with the concept of “strengthening healthy and eliminating pathogenic factors” and “regulating the balance of Yin and Yang” in TCM. This also underlies its wide clinical application and relatively low incidence of side effects.

### 3.3 The regulation of intestinal microecology by AM and its derivatives

AM and its derivatives are regarded as promising prebiotics for maintaining intestinal health. They can exert direct or indirect impacts on immune tolerance through the modulation of gut microbiota composition, immune responses and barrier protection, as summarized in **Table 2**.

**Table 2:** Effects of AM and its derivatives on gut microbiota.

Models	AM and its derivatives	Disease	Alterations in the Intestinal microecology	Reference
Broiler chicken	APS	Intestinal barrier dysfunction	*Parabacteroides distasonis* and *Bacteroides uniformis*	(68)
Mice	AM extract	UC	*Lactobacillus* and *Akkermansia*	(69)
Human	FAPS	–	*Lactobacillus*, *E. faecalis*, *Brautobacterium*, *Shigella, Romboutsia*, and *Clostridium_sensu_stricto_1*	(70)
Mice	APS	UC	*Muribaculaceae, Prevotellaceae_UCG-* *001*, *Alistipes, Rikenellaceae_RC9_gut_group* and *Muribaculum*	(71)
Mice	APS-E1F1	Immuno suppressed	*Butyrate-producing bacteria*	(72)
Mice	APS	Immune injury	Linoleic acid and α-linolenic acid in polyunsaturated fatty acid	(73)
Broiler chickens	APS	Necrotic enteritis	*Romboutsia, Halomonas*, propionic acid, butyric acid, formononetin, taurine, cholic acid and equol	(74)
Mice	APS	Constipation	*Blautia*;acetate,butyrate,and propionate	(75)
Mice	AM and Curcuma aromatica Salisb.	Colon cancer	*Adlercreutzia, Lachnospiraceae_UCG-001*,and *Parvibacter*, *Citrobacter* and *Candidatus_Arthromitus*; deoxycholic acid, lithocholic acid and ursodeoxycholic acid	(76)
Mice	APS	Dampness stagnancy due to spleen deficiency	*Pseudoflavonifractor and Paraprevotella, Parasutterella, Parabacteroides, Clostridium XIVb, Oscillibacter, Butyricicoccus*, and *Dorea*	(77)

AM and its derivatives restructure the gut microbiota toward a beneficial state, enhancing commensals and suppressing pathogens. This modulation effectively promotes the restoration and maintenance of immune homeostasis in a variety of disease models (68–70). Research indicates that APS can restore dietary polysaccharide-induced intestinal barrier dysfunction by selectively enriching *Parabacteroides distasonis* and *Bacteroides uniformis*, thereby activating signaling pathways associated with intestinal barrier function (68). In another study, *Lactobacillus rhamnosus* was applied for APS fermentation, and fecal samples from healthy volunteers were incubated with fermented Astragalus polysaccharides (FAPS). The findings indicated that FAPS exerted a marked effect on the gut microbiota by remodeling its community structure and enriching beneficial bacterial populations, including *Lactobacillus*, *Enterococcus faecalis*, and *Brautobacterium*, while suppressing pathogenic genera such as *Shigella*, *Romboutsia*, and *Clostridium_sensu_stricto_1* (70).

AM and its derivatives have been reported to enhance the synthesis of short-chain fatty acids (SCFAs) and show the potential to maintain immune homeostasis in disease models by improving the integrity of the intestinal barrier and modulating immune responses (71). Butyrate, an important SCFA produced by gut microbial metabolism, is most closely related to immunomodulatory activity. Research indicated that oral administration of APS markedly increased butyrate production in the intestine of mice, effectively restored the structural integrity and diversity of gut flora induced by cyclophosphamide (72). APS can also ameliorate the disrupted ratio of linoleic acid to α-linolenic acid in polyunsaturated fatty acid metabolism and effectively alleviate the immune injury and intestinal mucosal damage induced by chemotherapy (73). Additionally, APS enhances key microbial metabolites (e.g., propionate, butyrate, equol) and host compounds (e.g., formononetin, taurine), but lowers levels of uric acid, L-arginine, and serotonin. This phenomenon may be associated with inflammatory responses and the equilibrium between Th17 and Treg (74). However, some studies revealed that in aged rats with constipation, APS enhanced the abundance of *Blautia* while diminished the concentrations of acetate, butyrate, and propionate, and accordingly regulated glycolysis/glucose *de novo* metabolism and pyruvate metabolism (75). Another interesting study found that *Astragalus mongholicus Bunge* and *Curcuma aromatica Salisb* effectively inhibited CRC growth and reduced histological damage by regulating the flora structure to enhance the levels of beneficial bacteria like *Adlercreutzia* and reduce pathogens like Citrobacter, while also changing the bile acid composition (76). It may be related to the enrichment of specific bacteria and the up-regulation of FabG and baiA genes to promote the metabolic transformation of lithocholic acid. In summary, APS may trigger beneficial metabolic reprogramming of intestinal epithelial cells through the “microbiota-metabolite” axis. AM and its derivatives can reshape the structure of gut microbial and promote the conversion of ingested substances into specific metabolites (such as SCFAs, secondary bile acids, indole derivatives, etc.). These metabolites act as signaling molecules and energy substances, combining with local cells or remote tissues to regulate immune responses, thereby restoring the immune disorders caused by diseases (77, 78).

Current studies mainly concentrate on the direct effects of AM and its derivatives on the composition and metabolites of gut microbiota. Its potential antimicrobial activity and specific modulation of pathogenic microbes play a pivotal role in reshaping a healthy gut microecology and maintaining flora homeostasis. These microbiota-level alterations inevitably influence the host’s ultimate physiological or pathological phenotypes. Collectively, the mechanisms underlying the effects of AM and its derivatives on the digestive system can be consolidated into three synergistic dimensions. First, AM establishes a localized structural basis for immunomodulation by fortifying epithelial barrier function and regulating GALT responses within the intestine’s mucosal immune microenvironment. Second, AM and its derivatives achieve a precise equilibrium between suppressing hyperinflammation and maintaining immune tolerance by directly modulating immune cells. Third, indirect modulation of host immune homeostasis is exerted via the ‘microbiome-immunity axis’ through remodeling of gut microbiota composition and metabolism. In conclusion, AM acts as a “bridge” between the gut microbiota and the host system, providing a coherent mechanistic explanation for the “biphasic regulation” characteristics observed in digestive diseases.

## 4 Application of AM and its derivatives in digestive diseases

The digestive system possesses a unique mucosal immune structure that fulfills a critical function in preserving immune homeostasis and fostering immune tolerance. As a TCM with a long history, AM exhibits clear strengths in managing digestive ailments owing to its multi-component and multi-target features. Notably, AM and its extracts can produce therapeutic effects through different aspects in the treatment of IBD, CRC and gastric carcinoma, among others.

IBD is a chronic recurrent autoimmune disorder with unknown etiology. It represents one of the most challenging disorders of the gastrointestinal, mainly including UC and Crohn’s disease (79, 80). These disorders often arise when the immune system erroneously targets autologous tissues, resulting in inflammation and tissue injury. This process often involves dysregulated responses from various immune cells and cytokines (81–83). The main pathological mechanisms of the disease include the destruction of immune tolerance, the production of specific autoantigens, the activation of immune cells, and the dysregulated expression of cytokines (84–86). At present, most drugs used in the clinic have immunosuppressive effects, which could heighten the susceptibility to infection and cancer while ameliorating IBD. AM has been widely studied as a natural medicine, which plays an immunomodulatory role by enhancing the function of macrophages and T cells and controlling cytokine production to modulate immune responses, thereby alleviating symptoms associated with autoimmune diseases (87–89).

Gastrointestinal cancer is a diverse group of malignant tumors, mainly including esophageal cancer, gastric carcinoma, liver cancer, pancreatic cancer, and CRC (90). The development of these tumors involves intricate mechanisms, usually related to genetic factors, external influences, dietary habits, infection and other factors. For instance, Helicobacter pylori is a critical driver of gastric carcinogenesis, whereas CRC is closely linked to age, family history, dietary habits and certain genetic syndromes (91–93). In addition, the tumor microenvironment (TME) is instrumental in their development. Tumor cells coordinately regulate proliferation, invasion and distant metastasis via the interaction with surrounding stromal cells and immune cells (94, 95). AM and its derivatives can play an anti-cancer role by inducing apoptosis, modulating autophagy, inhibiting epithelial-mesenchymal metastasis, regulating immune response, and remodeling TME (96). Meanwhile, AM and its derivatives possess the potential to enhance the therapeutic efficacy of chemotherapeutic agents while alleviating their side effects, such as gastrointestinal discomforts including nausea and vomiting (97).

To date, the therapeutic potential of AM and its derivatives is strongly supported in IBD, CRC, and gastric cancer by extensive preclinical evidence; while the lack of robust clinical evidence in humans greatly constrains clinical translation. Currently, some studies suggest that the Huangqi Sijunzi Decoction can improve chemosensitivity in advanced gastric cancer by modulating IL-8 levels, whereas *Astragalus* injection combined with parenteral nutrition can facilitate postoperative recovery (98, 99). However, existing clinical studies on gastric cancer are largely limited by small sample sizes and inadequate design of blinding and control settings; Meanwhile, high-quality and large-scale randomized controlled trial (RCT) evidence for its application in IBD and CRC is still lacking. Although direct clinical evidence in gastroenterological diseases remains scarce, the well-documented clinical efficacy of AM in other conditions supports its broad therapeutic application prospect. For example, APS combined with adjuvant chemotherapy can alleviate chemotherapy-associated adverse effects and improve the health status of premenopausal breast cancer patients (100). Moreover, the results of a multicenter, evaluation-blinded, RCT in chronic kidney disease showed that the addition of AM therapy to standard therapy further stabilized renal function (101). AM and its derivatives have also shown promising preliminary clinical effects in the treatment of urinary tract infections and the prevention of chemotherapy-induced cardiotoxicity (102, 103). Collectively, existing clinical data highlight the promising future application prospects of AM and its derivatives.

## 5 Safety assessment and potential risks of AM and its derivatives

As a natural botanical, AM has demonstrated favorable safety and tolerability in both long-term traditional use and modern clinical investigations. Existing clinical evidence mainly focuses on compound formulations; for instance, the Huangqi Jianzhong Decoction exhibits therapeutic effects in Western medicine for chronic superficial gastritis without obvious adverse events (104). Furthermore, Cheng et al. reported that AM-containing formulas combined with platinum-based chemotherapy reduced treatment-related toxicity in patients with advanced gastric cancer (105).

While AM demonstrates favorable safety in polyherbal formulations, substantial heterogeneity exists among the toxicological profiles of its principal constituents: APS is characterized by a well-defined safety margin (106), AS-IV poses distinct developmental toxicity risks (107), yet safety data for flavonoids remain critically scarce. This underscores the necessity of transitioning the safety assessment of AM from the ‘whole herb’ level to the ‘component-specific’ level. Consequently, future safety evaluations and clinical guidance must account for this compositional heterogeneity to promote rational pharmacotherapy based on precise toxicological evidence.

AM demonstrates significant potential in specific combinational therapies; for instance, its co-administration with Metformin exerts synergistic efficacy and attenuates toxicity in diabetes management, while combination with chemotherapeutic regimens for colon cancer reduces the incidence of gastrointestinal adverse events (108, 109). Nevertheless, the extensive pharmacological activities of AM also raise potential risks in combinational therapy, mainly due to its inhibitory effects on major drug-metabolizing enzymes (CYP3A4 and CYP1A2) and the efflux transporter P-glycoprotein (110, 111). Such inhibition can alter the pharmacokinetic disposition of co-administered drugs metabolized via these pathways, potentially resulting in supratherapeutic plasma concentrations and subsequent toxicities. Therefore, clinicians should exercise caution, screen for overlapping metabolic pathways, and adopt therapeutic drug monitoring to ensure safe combinational therapy.

## 6 Conclusion and perspectives

AM is an important medicinal material in TCM. Its major active ingredients, including APS, AS and AF, have been widely investigated, especially for autoimmune disorders and tumors associated with the digestive system. A comprehensive review of current evidence indicates that AM and its derivatives can pull the disordered immune state back to a normal balance state by regulating the immune variety of cells as well as the intestinal microecology. This dual regulatory mechanism of AM and its derivatives forms a critical basis for developing therapies against conditions associated with immune dysregulation, such as cancer, autoimmune diseases, infection, and metabolic disorders.

Although AM has shown great potential in clinical application and mechanism research, it still faces multiple challenges to fully leverage its bidirectional immunomodulatory effect. To address these challenges and accelerate the translation of therapeutic potential into clinical benefits, this review systematically summarizes the available literature. First, we summarize the pharmacological effects of distinct AM components in **Table 1**, aiming to provide useful information for further studies. Current research on AM and its derivativesmainly focus on digestive system disorders, particularly CRC, IBD, and diarrhea. Current evidence indicates that specific bioactive fractions exhibit unique therapeutic priorities: polysaccharides are effective in modulating immune homeostasis, flavonoids are prominent for mitigating oxidative stress, and saponins demonstrate superior efficacy in anti-inflammatory responses. We note that current studies mainly focus on the functional mechanisms of AM components, while research on their structural characteristics remains limited, which may be the key to restricting their further validation. Furthermore, there is no clear standard for the extraction method, dose, and cycle of AM, which makes it difficult to ensure its repeatability. Secondly, this study systematically delineates the immunomodulatory mechanisms of AM using schematic diagrams: **Figure 1** illustrates the structural and functional basis of mucosal immunity in the digestive system, **Figure 2** elucidates the direct modulation of immune cells and their functions by AM and its derivatives, while **Table 2** summarizes the key regulatory effects on the gut microbiota. Numerous studies have validated the interactions between AM and its derivatives, intestinal microbiota and immune regulation, whereas the causal relationship between specific strains or their metabolites and immune regulation is still unknown. Third, an analysis of AM’s therapeutic applications indicates that the existing body of work is heavily skewed towards preclinical research. Robust evidence from large RCTs is still lacking to validate its safety and efficacy in diverse disease contexts. This represents the most critical challenge in transitioning from traditional empirical use and preclinical research to recognition by evidence-based medicine. Without overcoming this challenge, the efficacy, safety, optimal indications, and dosage of AM and its derivatives will continue to lack the high-grade evidence support that is widely accepted by the international medical community.

Conventional safety assessments of AM have predominantly focused on organ-level parameters, such as acute toxicity, hepatorenal function, and reproductive development. However, as a classic immunomodulator, the safety profile of AM is not merely reflected in the lack of direct cytotoxicity, but also lies in its ability to precisely regulate immune homeostasis, particularly the maintenance and enhancement of immune tolerance. AS-IV induces neurodevelopmental delays in offspring at doses devoid of overt maternal toxicity, suggesting a toxicity mechanism distinct from classical organ damage—likely involving disruption of the early neuro-endocrine-immune network. Although AS-IV exerts anti-inflammatory effects, its targeted immune pathways may overlap extensively with those critical for sustaining pregnancy tolerance; consequently, excessive or inappropriate immunosuppression may disrupt the delicate immunological equilibrium required for gestation. In contrast, APS facilitates immune tolerance via modulating immune homeostasis, which may account for its satisfactory clinical safety performance. Therefore, whether the developmental toxicity of AS-IV stems from its dysregulation of the immune-tolerant microenvironment during pregnancy represents a pivotal scientific question for future toxicological elucidation.

Future research in this field should prioritize clinical translation and practical application; however, translating AM into routine practice remains challenging. To address this issue, we propose the following strategies oriented toward clinical translation. First, to address the inherent variability in raw materials, processing, and formulation of AM, future research should systematically characterize the structural features of key components and elucidate the interaction mechanisms among multi-components and with other drugs. Advanced experimental models, including organoids, organ-on-a-chip, and immune-organoid co-culture systems, should be employed to investigate the effects of AM on intestinal immunity, barrier function, and microbiota metabolism. With high physiological relevance and throughput potential, these platforms can not only facilitate the intuitive validation of therapeutic efficacy by circumventing the limitations of traditional cell and animal models, but also support the development of personalized medicine via patient-specific modeling. Future studies should also focus on the integration of information technologies (e.g., network pharmacology, AI-assisted computational simulation, and high-throughput screening) with novel delivery systems such as nanoformulations and inclusion complexes, so as to enhance bioavailability and targeting efficiency. This integrated research strategy will facilitate the establishment of scientifically standardized quality control and pharmacokinetic evaluation systems, thereby promoting clinical translation and large-scale application of AM. Meanwhile, the interactions between AM and its derivatives and gut microbiota require further investigation to clarify dynamic changes in microbial composition and metabolites and their long-term effects on host immunity. Future studies should integrate multi-omics technologies, such as non-targeted metagenomics and serum/feces metabolomics, to systematically elucidate the intrinsic associations between functional modules involved in the intestinal barrier, immune responses, and systemic metabolism under AM modulation. To further establish the causal mechanisms of AM within the “microbiota-host” axis, future research should combine *in vivo* validation approaches, such as fecal microbiota transplantation and germ-free animal models, with the identification of key effector molecules. Clarifying the structure-function relationships of these components will facilitate significant breakthroughs in the mechanistic depth of related research. In addition, rigorous clinical trials are pivotal for verifying the results of preclinical studies and formulating standardized guidelines for the clinical application of AM and its derivatives for immunomodulation. Future clinical investigations should adopt mature research paradigms from other therapeutic areas to accelerate the clinical evaluation of AM and its derivatives in IBD, CRC, and other gastrointestinal diseases. Furthermore, future research should prioritize enhancing the clinical applicability and therapeutic development value of AM. For gastrointestinal diseases, RCTs should be designed to evaluate the combined regimen of “Western Medicine + AM,” assessing its synergistic effects on core clinical indicators, including symptom remission rate, recurrence rate, and quality of life. Furthermore, the clinical application value of AM in alleviating chemotherapy-induced vomiting and immunosuppressant-related hepatotoxicity remains to be further explored. It is anticipated that evidence-based AM regimens will be integrated into clinical pathways for digestive diseases, thereby standardizing their therapeutic application. The current deep integration of immunology, microbiology and pharmacology in this field provides broad prospects for translating these research results into personalized and effective therapies for immune-mediated diseases. Therefore, continuous interdisciplinary research and clinical verification are essential to fully unlock the therapeutic potential of AM and its derivatives in precise immune regulation.

## References

[1] Kenison JE Stevens NA Quintana FJ . Therapeutic induction of antigen-specific immune tolerance. Nat Rev Immunol. (2024) 24:338–57. doi: 10.1038/s41577-023-00970-x 38086932 PMC11145724

[2] Chen JY Shih LJ Liao MT Tsai KW Lu KC Hu WC . Understanding the immune system's intricate balance: Activation, tolerance, and self-protection. Int J Mol Sci. (2025) 26:5503. doi: 10.3390/ijms26125503 40564965 PMC12192980

[3] van Laar GG van Hamburg JP Tas SW . Extrathymic AIRE-expressing cells: Friends or foes in autoimmunity and cancer? Autoimmun Rev. (2022) 21:103141. doi: 10.1016/j.autrev.2022.103141 35840039

[4] Zheng P Dou Y Wang Q . Immune response and treatment targets of chronic hepatitis B virus infection: innate and adaptive immunity. Front Cell Infect Microbiol. (2023) 13:1206720. doi: 10.3389/fcimb.2023.1206720 37424786 PMC10324618

[5] Katakai T . Yin and yang roles of B lymphocytes in solid tumors: Balance between antitumor immunity and immune tolerance/immunosuppression in tumor-draining lymph nodes. Front Oncol. (2023) 13:1088129. doi: 10.3389/fonc.2023.1088129 36761946 PMC9902938

[6] Spahn TW Kucharzik T . Modulating the intestinal immune system: the role of lymphotoxin and GALT organs. Gut. (2004) 53:456–65. doi: 10.1136/gut.2003.023671 14960534 PMC1773987

[7] Nagler-Anderson C . Man the barrier! Strategic defences in the intestinal mucosa. Nat Rev Immunol. (2001) 1:59–67. doi: 10.1038/35095573 11905815

[8] Shukla A Chen C Jellusova J Leung CR Kao E Bhat N . Self-reactive B cells in the GALT are actively curtailed to prevent gut inflammation. JCI Insight. (2019) 5:e130621. doi: 10.1172/jci.insight.130621 31335327 PMC6777813

[9] Bemark M Pitcher MJ Dionisi C Spencer J . Gut-associated lymphoid tissue: a microbiota-driven hub of B cell immunity. Trends Immunol. (2024) 45:211–23. doi: 10.1016/j.it.2024.01.006 38402045 PMC11227984

[10] Zhang X Lin B Wang X Fang N Wu L Wan H . Research progress on the treatment of related diseases with Astragalus. Drug Des Devel Ther. (2025) 19:2845–62. doi: 10.2147/dddt.S494915 40248273 PMC12003202

[11] Zhu X Wei Y Dong J . A review on the pharmacological effects of Astragalus membranaceus in the treatment of bronchial asthma. J Basic Chin Med. (2021) 27:182–5.

[12] Ding Q Zu X Chen W Xin J Xu X Lv Y . Astragalus polysaccharide promotes the regeneration of intestinal stem cells through HIF-1 signalling pathway. J Cell Mol Med. (2024) 28:e18058. doi: 10.1111/jcmm.18058 38098246 PMC10844761

[13] Shi Y Shi X Zhao M Ma S Zhang Y . Pharmacological potential of Astragali Radix for the treatment of kidney diseases. Phytomedicine. (2024) 123:155196. doi: 10.1016/j.phymed.2023.155196 37952410

[14] Wang Y Zhang X Wang Y Zhao W Li H Zhang L . Application of immune checkpoint targets in the anti-tumor novel drugs and traditional Chinese medicine development. Acta Pharm Sin B. (2021) 11:2957–72. doi: 10.1016/j.apsb.2021.03.004 34729298 PMC8546663

[15] Meng Q Zeng F Hu Y Zheng Y Cheng F Wang Q . Textual research on the dosage of Astragalus membranaceus in Synopsis of Golden Chamberand discussion on its dose-effect relationship. China J Traditional Chin Med Pharm. (2024) 39:4055–8.

[16] Su HF Shaker S Kuang Y Zhang M Ye M Qiao X . Phytochemistry and cardiovascular protective effects of Huang-Qi (Astragali Radix). Med Res Rev. (2021) 41:1999–2038. doi: 10.1002/med.21785 33464616

[17] Qin ZT Wu ZH Wang CM Xie XC Wang YH . Astragalus polysaccharide as a potential antitumor immunomodulatory drug (Review). Mol Med Rep. (2025) 32:341. doi: 10.3892/mmr.2025.13706 41070602 PMC12522034

[18] Ercan L Akan H Çalışkan CG . Bioactive profile, anticarcinogenic, antimicrobial, antidiabetic effects, and in silico pharmacokinetic properties of Astragalus elatus. Bioorg Chem. (2025) 163:108733. doi: 10.1016/j.bioorg.2025.108733 40664154

[19] Hao J Hu R Zhao J Li Y Li Q Zhang X . Metabolomics combined with network pharmacology reveals the protective effect of astragaloside IV on alcoholic liver disease. Phytomedicine. (2024) 135:156032. doi: 10.1016/j.phymed.2024.156032 39270570

[20] Li S Hu X Liu F Hu W . Bioactive components and clinical potential of Astragalus species. Front Pharmacol. (2025) 16:1585697. doi: 10.3389/fphar.2025.1585697 40453655 PMC12122460

[21] Klichkhanov NK Suleimanova MN . Chemical composition and therapeutic effects of several Astragalus species (Fabaceae). Dokl Biol Sci. (2024) 518:172–86. doi: 10.1134/s0012496624701096 39128957

[22] Dong M Li J Yang D Li M Wei J . Biosynthesis and pharmacological activities of flavonoids, triterpene saponins and polysaccharides derived from Astragalus membranaceus. Molecules. (2023) 28:5018. doi: 10.3390/molecules28135018 37446680 PMC10343288

[23] Abd Elrahim Abd Elkader HT Essawy AE Al-Shami AS . Astragalus species: Phytochemistry, biological actions and molecular mechanisms underlying their potential neuroprotective effects on neurological diseases. Phytochemistry. (2022) 202:113293. doi: 10.1016/j.phytochem.2022.113293 35780924

[24] Sha W Zhao B Wei H Yang Y Yin H Gao J . Astragalus polysaccharide ameliorates vascular endothelial dysfunction by stimulating macrophage M2 polarization via potentiating Nrf2/HO-1 signaling pathway. Phytomedicine. (2023) 112:154667. doi: 10.1016/j.phymed.2023.154667 36842218

[25] Wei X Xin J Chen W Wang J Lv Y Wei Y . Astragalus polysaccharide ameliorated complex factor-induced chronic fatigue syndrome by modulating the gut microbiota and metabolites in mice. BioMed Pharmacother. (2023) 163:114862. doi: 10.1016/j.biopha.2023.114862 37167729

[26] Li Q Zhang C Xu G Shang X Nan X Li Y . Astragalus polysaccharide ameliorates CD8(+) T cell dysfunction through STAT3/Gal-3/LAG3 pathway in inflammation-induced colorectal cancer. BioMed Pharmacother. (2024) 171:116172. doi: 10.1016/j.biopha.2024.116172 38278025

[27] Zhong Y Xiao Q Kang Z Huang J Ge W Wan Q . Astragalus polysaccharide alleviates ulcerative colitis by regulating the balance of Tfh/Treg cells. Int Immunopharmacol. (2022) 111:109108. doi: 10.1016/j.intimp.2022.109108 35926271

[28] Ye M Fan M Zhao Y Wang F Yang X Yao W . Low molecular weight Astragalus membranaceus polysaccharides alleviates dextran sulfate sodium-induced colitis in mice. Carbohydr Polym. (2025) 367:124050. doi: 10.1016/j.carbpol.2025.124050 40817502

[29] Liu YX Song XM Dan LW Tang JM Jiang Y Deng C . Astragali Radix: comprehensive review of its botany, phytochemistry, pharmacology and clinical application. Arch Pharm Res. (2024) 47:165–218. doi: 10.1007/s12272-024-01489-y 38493280

[30] Yang L Han X Xing F Wu H Shi H Huang F . Total flavonoids of astragalus attenuates experimental autoimmune encephalomyelitis by suppressing the activation and inflammatory responses of microglia via JNK/AKT/NFκB signaling pathway. Phytomedicine. (2021) 80:153385. doi: 10.1016/j.phymed.2020.153385 33091854

[31] Xia H He W Lv C Zhang J Lin X Qin S . The inhibitory effect of Astragalus flavone extract on hyperuricemia and its underlying molecular mechanism by targeting JNK/AP-1/NLRP3/IL-1β signaling pathway. Phytomedicine. (2025) 140:156622. doi: 10.1016/j.phymed.2025.156622 40073779

[32] Chen L Kong X Zhou R Hu J Zhou R Song Z . Proteomics reveals the pharmacological mechanism of flavonoids from Astragali Complanati Semen in preventing chronic liver injury. Phytomedicine. (2024) 133:155910. doi: 10.1016/j.phymed.2024.155910 39059265

[33] Liu Y Zhou M Mu Q Qi J An X . Research progress on biological functions of Astragalus flavonoids and their application in animal production. Feed Res. (2026) 49:144–9. doi: 10.13557/j.cnki.issn1002-2813.2026.06.024

[34] Che Y Li L Kong M Geng Y Wang D Li B . Dietary supplementation of Astragalus flavonoids regulates intestinal immunology and the gut microbiota to improve growth performance and intestinal health in weaned piglets. Front Immunol. (2024) 15:1459342. doi: 10.3389/fimmu.2024.1459342 39416777 PMC11479930

[35] Cao S Lv B Tai Y Zuo HX Xing Y Surh YJ . Formononetin ameliorates DSS-induced colitis by inhibiting the MAPK/PPAR-γ/NF-κB/ROS signaling pathways. Toxicol Appl Pharmacol. (2025) 496:117239. doi: 10.1016/j.taap.2025.117239 39855309

[36] Liu M Yang C Peng X Zheng S He H Wang W . Formononetin suppresses colitis-associated colon cancer by targeting lipid synthesis and mTORC2/Akt signaling. Phytomedicine. (2025) 142:156665. doi: 10.1016/j.phymed.2025.156665 40318528

[37] Salehi B Carneiro JNP Rocha JE Coutinho HDM Morais Braga MFB Sharifi-Rad J . Astragalus species: Insights on its chemical composition toward pharmacological applications. Phytother Res. (2021) 35:2445–76. doi: 10.1002/ptr.6974 33325585

[38] Tian L Zhao JL Kang JQ Guo SB Zhang N Shang L . Astragaloside IV alleviates the experimental DSS-induced colitis by remodeling macrophage polarization through STAT signaling. Front Immunol. (2021) 12:740565. doi: 10.3389/fimmu.2021.740565 34589089 PMC8473681

[39] Zhang X Zhang F Li Y Fan N Zhao K Zhang A . Blockade of PI3K/AKT signaling pathway by Astragaloside IV attenuates ulcerative colitis via improving the intestinal epithelial barrier. J Transl Med. (2024) 22:406. doi: 10.1186/s12967-024-05168-w 38689349 PMC11061986

[40] Wen LP Gao SW Chen HX Liu Q Xiao GZ Lin HC . Astragaloside IV ameliorates colonic adenomatous polyps development by orchestrating gut Bifidobacterium and serum metabolome. Am J Chin Med. (2024) 52:1527–54. doi: 10.1142/s0192415x24500605 39164214

[41] Sun L Zhang B . The digestive system and autoimmunity. BMC Immunol. (2023) 24:36. doi: 10.1186/s12865-023-00561-4 37794375 PMC10552442

[42] Neurath MF Artis D Becker C . The intestinal barrier: a pivotal role in health, inflammation, and cancer. Lancet Gastroenterol Hepatol. (2025) 10:573–92. doi: 10.1016/s2468-1253(24)00390-x 40086468

[43] Liebing E Krug SM Neurath MF Siegmund B Becker C . Wall of resilience: How the intestinal epithelium prevents inflammatory onslaught in the gut. Cell Mol Gastroenterol Hepatol. (2025) 19:101423. doi: 10.1016/j.jcmgh.2024.101423 39461590 PMC11720114

[44] Gustafsson JK Johansson MEV . The role of goblet cells and mucus in intestinal homeostasis. Nat Rev Gastroenterol Hepatol. (2022) 19:785–803. doi: 10.1038/s41575-022-00675-x 36097076

[45] Mörbe UM Jørgensen PB Fenton TM von Burg N Riis LB Spencer J . Human gut-associated lymphoid tissues (GALT); diversity, structure, and function. Mucosal Immunol. (2021) 14:793–802. doi: 10.1038/s41385-021-00389-4 33753873

[46] Torow N Li R Hitch TCA Mingels C Al Bounny S van Best N . M cell maturation and cDC activation determine the onset of adaptive immune priming in the neonatal Peyer's patch. Immunity. (2023) 56:1220–1238.e7. doi: 10.1016/j.immuni.2023.04.002 37130522 PMC10262694

[47] Jain S Bemark M Spencer J . Human gut-associated lymphoid tissue: A dynamic hub propagating modulators of inflammation. Clin Transl Med. (2023) 13:e1417. doi: 10.1002/ctm2.1417 37735775 PMC10514258

[48] Shaikh H Vargas JG Mokhtari Z Jarick KJ Ulbrich M Mosca JP . Mesenteric lymph node transplantation in mice to study immune responses of the gastrointestinal tract. Front Immunol. (2021) 12:689896. doi: 10.3389/fimmu.2021.689896 34381447 PMC8352558

[49] Luu K Ye JY Lagishetty V Liang F Hauer M Sedighian F . Fecal and tissue microbiota are associated with tumor T-cell infiltration and mesenteric lymph node involvement in colorectal cancer. Nutrients. (2023) 15:316. doi: 10.3390/nu15020316 36678187 PMC9861998

[50] Yeh CL Wu JM Chen KY Wu MH Yang PJ Lee PC . Effects of different routes and forms of vitamin D administration on mesenteric lymph node CD4+ T cell polarization and intestinal injury in obese mice complicated with polymicrobial sepsis. Nutrients. (2022) 14:3557. doi: 10.3390/nu14173557 36079813 PMC9460651

[51] Zhang Q Zeng Z Wei N Su Y Wang J Ni Q . Mesenteric lymph nodes: a critical site for the up-regulatory effect of hUC-MSCs on Treg cells by producing TGF-β1 in colitis treatment. Stem Cell Res Ther. (2024) 15:190. doi: 10.1186/s13287-024-03809-x 38956621 PMC11218300

[52] Lockhart A Mucida D Bilate AM . Intraepithelial lymphocytes of the intestine. Annu Rev Immunol. (2024) 42:289–316. doi: 10.1146/annurev-immunol-090222-100246 38277691 PMC11608099

[53] Ceglia S Berthelette A Howley K Li Y Mortzfeld B Bhattarai SK . An epithelial cell-derived metabolite tunes immunoglobulin A secretion by gut-resident plasma cells. Nat Immunol. (2023) 24:531–44. doi: 10.1038/s41590-022-01413-w 36658240 PMC10243503

[54] Koboziev I Karlsson F Grisham MB . Gut-associated lymphoid tissue, T cell trafficking, and chronic intestinal inflammation. Ann N Y Acad Sci. (2010) 1207 Suppl 1:E86–93. doi: 10.1111/j.1749-6632.2010.05711.x 20961311 PMC3075575

[55] Wang D Cui Q Yang YJ Liu AQ Zhang G Yu JC . Application of dendritic cells in tumor immunotherapy and progress in the mechanism of anti-tumor effect of Astragalus polysaccharide (APS) modulating dendritic cells: a review. BioMed Pharmacother. (2022) 155:113541. doi: 10.1016/j.biopha.2022.113541 36127221

[56] An EK Zhang W Kwak M Lee PC Jin JO . Polysaccharides from Astragalus membranaceus elicit T cell immunity by activation of human peripheral blood dendritic cells. Int J Biol Macromol. (2022) 223:370–7. doi: 10.1016/j.ijbiomac.2022.11.048 36368354

[57] Shen J Zhao Y Cui W . Astragalus mongholicus Bunge extract improves ulcerative colitis by promoting PLCB2 to inhibit colonic epithelial cell pyroptosis. J Ethnopharmacol. (2024) 334:118554. doi: 10.1016/j.jep.2024.118554 38992398

[58] Ying Y Song LY Pang WL Zhang SQ Yu JZ Liang PT . Astragalus polysaccharide protects experimental colitis through an aryl hydrocarbon receptor-dependent autophagy mechanism. Br J Pharmacol. (2024) 181:681–97. doi: 10.1111/bph.16229 37653584

[59] Bamodu OA Kuo KT Wang CH Huang WC Wu ATH Tsai JT . Astragalus polysaccharides (PG2) enhances the M1 polarization of macrophages, functional maturation of dendritic cells, and T cell-mediated anticancer immune responses in patients with lung cancer. Nutrients. (2019) 11:2264. doi: 10.3390/nu11102264 31547048 PMC6836209

[60] Liu D Zhu Y Hou Z Wang H Li Q . Polysaccharides from Astragalus membranaceus Bunge alleviate LPS-induced neuroinflammation in mice by modulating microbe-metabolite-brain axis and MAPK/NF-κB signaling pathway. Int J Biol Macromol. (2025) 304:140885. doi: 10.1016/j.ijbiomac.2025.140885 39938846

[61] Li Y Yu P Fu W Cai L Yu Y Feng Z . Ginseng-Astragalus-oxymatrine injection ameliorates cyclophosphamide-induced immunosuppression in mice and enhances the immune activity of RAW264.7 cells. J Ethnopharmacol. (2021) 279:114387. doi: 10.1016/j.jep.2021.114387 34216728

[62] Li S Dou B Shu S Wei L Zhu S Ke Z . Suppressing NK cells by Astragaloside IV protects against acute ischemic stroke in mice via inhibiting STAT3. Front Pharmacol. (2021) 12:802047. doi: 10.3389/fphar.2021.802047 35185544 PMC8852846

[63] Yakubogullari N Cagir A Bedir E Sag D . Astragalus saponins, Astragaloside VII and newly synthesized derivatives, induce dendritic cell maturation and T cell activation. Vaccines (Basel). (2023) 11:495. doi: 10.3390/vaccines11030495 36992079 PMC10059926

[64] Liu P Wang S Bin Y Xin Z Yang H Zhang T . Astragalus polysaccharide promotes CD8 + T cell activity by downregulating Tim-3 to potentiate antitumor immunity. Mol Immunol. (2025) 188:121–30. doi: 10.1016/j.molimm.2025.11.010 41270296

[65] Sun P Zhu L Yu Y Hu S Shan M Zhao X . Combination of Astragalus-Salvia and Ophiopogon-Dendrobium herb pairs alleviates Sjögren's syndrome via inhibiting the JAK1/STAT3 and PI3K/AKT pathways in NOD/Ltj mice. Chin J Nat Med. (2025) 23:733–41. doi: 10.1016/s1875-5364(25)60892-2 40545318

[66] Li Z Sun Q Liu Q Mu X Wang H Zhang H . Compound 511 ameliorates MRSA-induced lung injury by attenuating morphine-induced immunosuppression in mice via PI3K/AKT/mTOR pathway. Phytomedicine. (2023) 108:154475. doi: 10.1016/j.phymed.2022.154475 36252465

[67] Xu N Han X Zhang X Wang J Yuan J Wang M . Huangqi-Guizhi-Wuwu decoction regulates differentiation of CD4(+) T cell and prevents against experimental autoimmune encephalomyelitis progression in mice. Phytomedicine. (2024) 125:155239. doi: 10.1016/j.phymed.2023.155239 38308917

[68] Yang J Sun Y Wang Q Yu S Li Y Yao B . Astragalus polysaccharides-induced gut microbiota play a predominant role in enhancing of intestinal barrier function of broiler chickens. J Anim Sci Biotechnol. (2024) 15:106. doi: 10.1186/s40104-024-01060-1 39103958 PMC11302362

[69] Zhu J Shentu C Meng Q Fan S Tang Y Mao M . Astragalus membranaceus extract attenuates ulcerative colitis by integrating multiomics and the PI3K/AKT signaling pathway. Front Pharmacol. (2025) 16:1585748. doi: 10.3389/fphar.2025.1585748 40552164 PMC12183238

[70] Yang P Zhou Q Zhang Y Jia M Li R Qu Q . Exploring the prebiotic potential of fermented Astragalus polysaccharides on gut microbiota regulation *in vitro*. Curr Microbiol. (2024) 82:52. doi: 10.1007/s00284-024-04035-7 39709319

[71] Zhang Y Ji W Qin H Chen Z Zhou Y Zhou Z . Astragalus polysaccharides alleviate DSS-induced ulcerative colitis in mice by restoring SCFA production and regulating Th17/Treg cell homeostasis in a microbiota-dependent manner. Carbohydr Polym. (2025) 349:122829. doi: 10.1016/j.carbpol.2024.122829 39643403

[72] Rong X Shu Q . Enhancing immunomodulation in cyclophosphamide-induced immunosuppressed mice through targeted modulation of butyrate-producing gut microbiota via oral administration of astragalus polysaccharides. Food Sci Nutr. (2024) 12:7683–95. doi: 10.1002/fsn3.4386 39479666 PMC11521734

[73] Wang H Zhu W Hong Y Wei W Zheng N He X . Astragalus polysaccharides attenuate chemotherapy-induced immune injury by modulating gut microbiota and polyunsaturated fatty acid metabolism. Phytomedicine. (2024) 128:155492. doi: 10.1016/j.phymed.2024.155492 38479258

[74] Song B Li P Yan S Liu Y Gao M Lv H . Effects of dietary Astragalus polysaccharide supplementation on the Th17/Treg balance and the gut microbiota of broiler chickens challenged with necrotic enteritis. Front Immunol. (2022) 13:781934. doi: 10.3389/fimmu.2022.781934 35265068 PMC8899652

[75] Liu X Li M Jian C Wei F Liu H Li K . Astragalus polysaccharide alleviates constipation in the elderly via modification of gut microbiota and fecal metabolism. Rejuvenation Res. (2022) 25:275–90. doi: 10.1089/rej.2022.0039 36205566

[76] Wang X Zhu B Hua Y Sun R Tan X Chang X . Astragalus mongholicus Bunge and Curcuma aromatica Salisb. modulate gut microbiome and bile acid metabolism to inhibit colon cancer progression. Front Microbiol. (2024) 15:1395634. doi: 10.3389/fmicb.2024.1395634 38952445 PMC11215047

[77] Zhao W Duan C Liu Y Lu G Lyu Q Liu X . Modulating effects of Astragalus polysaccharide on immune disorders via gut microbiota and the TLR4/NF-κB pathway in rats with syndrome of dampness stagnancy due to spleen deficiency. J Zhejiang Univ-Sci B. (2023) 24:650–62. doi: 10.1631/jzus.B2200491 37455140 PMC10350370

[78] Tyagi A Kumar V . The gut microbiota-bile acid axis: a crucial regulator of immune function and metabolic health. World J Microbiol Biotechnol. (2025) 41:215. doi: 10.1007/s11274-025-04395-7 40555888

[79] Saez A Herrero-Fernandez B Gomez-Bris R Sánchez-Martinez H Gonzalez-Granado JM . Pathophysiology of inflammatory bowel disease: innate immune system. Int J Mol Sci. (2023) 24:1526. doi: 10.3390/ijms24021526 36675038 PMC9863490

[80] Luo M Zhao F Cheng H Su M Wang Y . Macrophage polarization: an important role in inflammatory diseases. Front Immunol. (2024) 15:1352946. doi: 10.3389/fimmu.2024.1352946 38660308 PMC11039887

[81] Fu X Liu H Huang G Dai SS . The emerging role of neutrophils in autoimmune-associated disorders: effector, predictor, and therapeutic targets. MedComm (2020). (2021) 2:402–13. doi: 10.1002/mco2.69 34766153 PMC8554667

[82] Zhang X Mei D Zhang L Wei W . Src family protein kinase controls the fate of B cells in autoimmune diseases. Inflammation. (2021) 44:423–33. doi: 10.1007/s10753-020-01355-1 33037966

[83] Zhang P Lu Q . Genetic and epigenetic influences on the loss of tolerance in autoimmunity. Cell Mol Immunol. (2018) 15:575–85. doi: 10.1038/cmi.2017.137 29503444 PMC6079019

[84] Hu D Murugaiyan G . CD8(+) Tregs kill pathogenic cells to avert autoimmunity. Trends Immunol. (2022) 43:415–6. doi: 10.1016/j.it.2022.04.006 35527183

[85] Bluestone JA Bour-Jordan H Cheng M Anderson M . T cells in the control of organ-specific autoimmunity. J Clin Invest. (2015) 125:2250–60. doi: 10.1172/jci78089 25985270 PMC4497750

[86] Faas PPM Scharmann SD Pishesha N . Antigen-specific tolerance: clinical and preclinical approaches in autoimmunity. Eur J Immunol. (2025) 55:e70067. doi: 10.1002/eji.70067 41054017

[87] Zeng Y Cao W Huang Y Zhang H Li C He J . Huangqi Baihe granules alleviate hypobaric hypoxia-induced acute lung injury in rats by suppressing oxidative stress and the TLR4/NF-κB/NLRP3 inflammatory pathway. J Ethnopharmacol. (2024) 324:117765. doi: 10.1016/j.jep.2024.117765 38228230

[88] Chen Z Liu L Gao C Chen W Vong CT Yao P . Astragali Radix (Huangqi): a promising edible immunomodulatory herbal medicine. J Ethnopharmacol. (2020) 258:112895. doi: 10.1016/j.jep.2020.112895 32330511

[89] Huo Z Li J Li X Xiao H Lin Y Ma Y . Functional fractions of Astragalus polysaccharides as a potential prebiotic to alleviate ulcerative colitis. Int J Biol Macromol. (2024) 271:132580. doi: 10.1016/j.ijbiomac.2024.132580 38788871

[90] Abdul-Latif M Townsend K Dearman C Shiu KK Khan K . Immunotherapy in gastrointestinal cancer: the current scenario and future perspectives. Cancer Treat Rev. (2020) 88:102030. doi: 10.1016/j.ctrv.2020.102030 32505807

[91] Li Y Liu G Zhou L Wang Y Sun Y Chen Y . Helicobacter pylori-induced apoptosis in gastric diseases: mechanisms, implications, and diagnostic applications. Int J Gen Med. (2025) 18:2995–3009. doi: 10.2147/ijgm.S520982 40524752 PMC12168956

[92] Zhou J Yang Q Zhao S Sun L Li R Wang J . Evolving landscape of colorectal cancer: global and regional burden, risk factor dynamics, and future scenarios (the Global Burden of Disease 1990-2050). Ageing Res Rev. (2025) 104:102666. doi: 10.1016/j.arr.2025.102666 39828028

[93] Ma Y Ni J Mei P Chen Y Guo X . The burden of colorectal cancer attributable to diet low in whole grains from 1990 to 2021: a global, regional and national analysis. Front Nutr. (2025) 12:1527522. doi: 10.3389/fnut.2025.1527522 40271437 PMC12014444

[94] Wang B Tan B . Noncoding RNAs: regulating the crosstalk between tumor-associated macrophages and gastrointestinal cancer. BioMed Pharmacother. (2022) 153:113370. doi: 10.1016/j.biopha.2022.113370 35792392

[95] Xu Z Chen Y Ma L Chen Y Liu J Guo Y . Role of exosomal non-coding RNAs from tumor cells and tumor-associated macrophages in the tumor microenvironment. Mol Ther. (2022) 30:3133–54. doi: 10.1016/j.ymthe.2022.01.046 35405312 PMC9552915

[96] Wang W Zhou H Sen A Zhang P Yuan L Zhou S . Recent advances in the mechanisms and applications of Astragalus polysaccharides in liver cancer treatment: an overview. Molecules. (2025) 30:2792. doi: 10.3390/molecules30132792 40649307 PMC12250682

[97] Zhang X Qiu H Li C Cai P Qi F . The positive role of traditional Chinese medicine as an adjunctive therapy for cancer. Biosci Trends. (2021) 15:283–98. doi: 10.5582/bst.2021.01318 34421064

[98] Xie G He Y Zhai J Yao X Shen L . Clinical study on Astragalus Sijunzi decoction enhancing sensitivity of preoperative neoadjuvant chemotherapy for advanced gastric cancer. China J Traditional Chin Med Pharm. (2022) 37:1810–4.

[99] Jia S Qiao C Wu X . Clinical study on postoperative rehabilitation of gastric cancer patients with Astragalus injection combined with parenteral nutrition support. Chin J Clin Pharmacol. (2022) 38:99–102. doi: 10.13699/j.cnki.1001-6821.2022.02.001

[100] Shen WC Chen SC Wang CH Hung CM Peng MT Liu CT . Astragalus polysaccharides improve adjuvant chemotherapy-induced fatigue for patients with early breast cancer. Sci Rep. (2024) 14:25690. doi: 10.1038/s41598-024-76627-z 39465324 PMC11514294

[101] Chan KW Kwong ASK Tsui PN Chan GCW Choi WF Yiu WH . Add-on astragalus in type 2 diabetes and chronic kidney disease: a multi-center, assessor-blind, randomized controlled trial. Phytomedicine. (2024) 130:155457. doi: 10.1016/j.phymed.2024.155457 38810556

[102] Salvatore S Ruffolo AF Stabile G Casiraghi A Zito G De Seta F . A randomized controlled trial comparing a new D-mannose-based dietary supplement to placebo for the treatment of uncomplicated Escherichia coli urinary tract infections. Eur Urol Focus. (2023) 9:654–9. doi: 10.1016/j.euf.2022.12.013 36621376

[103] Li X Guo X Li J Yuan L Wang H . Preventing effect of astragalus polysaccharide on cardiotoxicity induced by chemotherapy of epirubicin: a pilot study. Med (Baltimore). (2022) 101:e30000. doi: 10.1097/md.0000000000030000 35960075 PMC9371539

[104] Huang J Xiong M . Clinical observation on Huangqi Jianzhong decoction in treating chronic superficial gastritis of spleen-stomach deficiency cold type. J Chin Medicinal Materials. (2022) 45:986–8. doi: 10.13863/j.issn1001-4454.2022.04.041

[105] Cheng M Hu J Zhao Y Jiang J Qi R Chen S . Efficacy and safety of astragalus-containing traditional Chinese medicine combined with platinum-based chemotherapy in advanced gastric cancer: a systematic review and meta-analysis. Front Oncol. (2021) 11:632168. doi: 10.3389/fonc.2021.632168 34422628 PMC8371531

[106] Li Q Li J Wang Y Wu F Li T . Efficacy and safety of astragalus polysaccharides in patients with Malignant tumors: a systematic review and meta-analysis. Naunyn Schmiedebergs Arch Pharmacol. (2025) 398:11705–32. doi: 10.1007/s00210-025-04074-2 40208321

[107] Xuying W Jiangbo Z Yuping Z Xili M Yiwen Z Tianbao Z . Effect of astragaloside IV on the general and peripartum reproductive toxicity in Sprague-Dawley rats. Int J Toxicol. (2010) 29:505–16. doi: 10.1177/1091581810376840 20884860

[108] Du Z Wu S Li H Li G Xu J . Study on compatibility rules of traditional Chinese medicine combined with metformin in the treatment of diabetes. Sci Technol Eng. (2023) 23:11145–56.

[109] Rong Y Zhang B Wu H Qiu H Hu Pili Qian S . Clinical observation on Astragalus polysaccharide for injection in alleviating side effects of chemotherapy in stage II colon cancer. J Chin Medicinal Materials. (2011) 34:657–9. doi: 10.13863/j.issn1001-4454.2011.04.047

[110] Wei X Leng X Liang J Liu J Chi L Deng H . Pharmacological potential of natural medicine Astragali Radix in treating intestinal diseases. BioMed Pharmacother. (2024) 180:117580. doi: 10.1016/j.biopha.2024.117580 39413615

[111] Wang T Chen X Gao Q Huang C Wang K Qiu F . Herb-drug interaction potential of Astragali Radix: a metabolic perspective. Drug Metab Rev. (2025) 57:9–25. doi: 10.1080/03602532.2024.2441235 39692050

[112] Chen Y Wang J Li J Zhu J Wang R Xi Q . Astragalus polysaccharide prevents ferroptosis in a murine model of experimental colitis and human Caco-2 cells via inhibiting NRF2/HO-1 pathway. Eur J Pharmacol. (2021) 911:174518. doi: 10.1016/j.ejphar.2021.174518 34562468

